# Extended Renal Outcomes with Use of Iodixanol versus Iohexol after Coronary Angiography

**DOI:** 10.1155/2014/506479

**Published:** 2014-08-07

**Authors:** Horng-Ruey Chua, Mark Horrigan, Elizabeth Mcintosh, Rinaldo Bellomo

**Affiliations:** ^1^Department of Intensive Care, Austin Hospital, Melbourne, VIC 3084, Australia; ^2^Division of Nephrology, University Medicine Cluster, National University Hospital, National University Health System, Singapore 119228; ^3^Department of Cardiology, Austin Hospital, Melbourne, VIC 3084, Australia; ^4^Australian and New Zealand Intensive Care Research Committee (ANZIC-RC), School of Public Health and Preventive Medicine, Monash University, Melbourne, VIC 3181, Australia

## Abstract

The impact of isoosmolar versus low-osmolar contrast media (CM) administration on contrast-induced acute kidney injury (CI-AKI) and extended renal dysfunction (ERD) is unclear. We retrospectively examined incidences of CI-AKI and ERD in patients who received iodixanol (isoosmolar) versus iohexol (low-osmolar) during angiography for cardiac indications. Of 713 patients, 560 (cohort A), 190 (cohort B), and 172 (cohort C) had serum creatinine monitored at 3 days, 30 days, and 6 months after angiography, respectively. 18% of cohort A developed CI-AKI, which was more common with iodixanol than iohexol (22% versus 13%, *P* = 0.006). However, patients given iodixanol were older with lower baseline estimated glomerular filtration rates (eGFR). On multivariate analysis, independent associations with higher CI-AKI risk include age >65 years, female gender, cardiac failure, ST-elevation myocardial infarction, intra-aortic balloon pump, and critical illness, but not CM type, higher CM load, or eGFR < 45 mL/min/1.73 m^2^. 32% of cohort B and 34% of cohort C had ERD at 30 days and 6 months, while 44% and 41% of subcohorts had ERD at 90 days and 1 year, respectively. CI-AKI, but not CM type, was associated with medium- and longer-term ERD, with 3-fold higher risk. Advanced age, emergent cardiac conditions, and critical illness are stronger predictors of CI-AKI, compared with CM-related factors. CI-AKI predicts longer-term ERD.

## 1. Introduction

Contrast media (CM) is the third most common cause of hospital-acquired acute kidney injury (AKI), and coronary angioplasty accounts for the highest incidence of contrast-induced AKI (CI-AKI) [1]. CI-AKI risk is exacerbated by comorbidities including advanced age, diabetes mellitus (DM), congestive cardiac failure (CCF), and chronic kidney disease (CKD) [2], all of which are highly prevalent in patients with coronary artery disease. Thus, numerous CI-AKI preventive strategies have been employed, such as reduced CM load and avoidance of recurrent exposure [3], intravascular volume expansion [4], N-acetylcysteine administration [5], and preferred use of isoosmolar CM (IOCM) or low-osmolar CM (LOCM) over high-osmolar CM (HOCM) [6].

In relation to CM, use of IOCM iodixanol has been reported to reduce CI-AKI risk in patients with DM and CKD, compared to LOCMs [7], in particular iohexol [8]. Therefore, clinicians may prefer to use iodixanol in high-risk subjects. However, findings on the apparent benefit of iodixanol over other LOCMs on renal function have not been consistent [9, 10]. Further conclusive comparisons are difficult due to highly variable definitions of CI-AKI and failure to account for confounding factors for AKI such as critical illness. Most studies also involved short-term follow-up and examined only acute renal dysfunction, while prognostication and knowledge of longer-term renal outcomes are scarce.

Therefore, we wish to evaluate the incidence of CI-AKI and the temporal evolution of renal function over one year, in patients who received iodixanol versus iohexol for intra-arterial angiography, using modern consensus definitions of AKI. We aim to identify the risk factors and prognostic implications of AKI in relation to different CM used, while accounting for confounding effects of CCF, CKD, and critical illness. We hypothesize that (i) use of iohexol versus iodixanol would be associated with a higher risk of CI-AKI and (ii) CI-AKI would, in turn, be associated with extended renal dysfunction (ERD) in medium-term survivors.

## 2. Methods

### 2.1. Study Design and Population

We performed a single-centre, retrospective observational study, on the use of iodixanol (IOCM) versus iohexol (LOCM) in patients who underwent emergency or elective intra-arterial angiography, in our tertiary institution from January till December 2009. The patient list and identifiers were retrieved from routine procedural records. The Human Research Ethics Committee approved the study and waived the need for informed consent. All patients aged >18 years were included. The exclusion criteria were (i) patients with end-stage renal disease (ESRD) on maintenance dialysis, (ii) patients with no follow-up renal function assessment after angiography, and (iii) patients whose contrast type was not specified.

### 2.2. Study Definitions and Data Collection

All baseline patient demographics and comorbidities were indexed at time of angiography and captured in the procedural database. Critical illness was defined as admission(s) to the intensive care unit (ICU) within one week of angiography. The date of angiography was defined as day 0 (D0). Baseline serum creatinine level (sCr) was the patients' lowest sCr performed from D-7 to D0. If this was not available, it was inferred from the* lowest* sCr available in our electronic health records, up to one year from angiography. Baseline estimated glomerular filtration rate (eGFR) was calculated using the 4-variable MDRD equation [11].* Peak* sCr were obtained during respective time-windows from initial contrast exposure: D1–D5, D20–D40, D70–D110, D150–D210, and D270–D450, as surrogates of renal function at 3 days, 30 days, 90 days, 6 months, and 1 year after angiography, respectively.

CI-AKI was defined as having fulfilled minimum “R” criterion (≥1.5x increase in peak sCr from baseline) of the RIFLE “at-risk/injury/failure (R/I/F)” classification [12] at 3 days after contrast. ERD was described at medium-term (30 days and 90 days) and longer-term (6 months and 1 year), having to fulfil minimum RIFLE “R” criterion at respective time-windows, which indicates failure to improve to baseline renal function. Corresponding delta-sCr levels were calculated from the difference between respective peak and baseline sCr.

### 2.3. Contrast and Angiography Details

We used iodixanol (Visipaque-320, GE Healthcare, Rydalmere, Australia) that contains 652 mg iodixanol/mL and 320 mg of elemental iodine and iohexol (Omnipaque-350, GE Healthcare, Rydalmere, Australia) that contains 755 mg iohexol/mL and 350 mg of elemental iodine. The iodine dose was calculated from (iodine concentration of respective CM × volume administered). The CM-load administered was defined as ratio of iodine dose infused corrected for baseline eGFR (grams iodine/eGFR), in view that iodine-dose to creatinine-clearance ratio correlates well with the area under CM concentration-time curve [13].

The primary indication for angiography was identified from procedural records, including ST-elevation myocardial infarction (STEMI) and non-STEMI (NSTEMI). Likewise, angiography details including coronary angiogram (COROS), percutaneous coronary intervention (PCI), aortogram, left ventriculogram (LVgram), and intra-aortic balloon pump (IABP) insertion were retrieved if performed.

### 2.4. Data Analysis

The study subjects were divided into three cohorts for univariate analysis. Cohorts A, B, and C included all patients with available sCr at 3 days, 30 days, and 6 months, respectively. Incidences of CI-AKI and ERD were determined at prespecified time-points, between patients administered iodixanol and iohexol, and described separately for each cohort. Parametric variables were presented in mean (± standard deviation) and nonparametric variables in median (interquartile range); univariate comparisons were then performed using Students' *t*-test and Wilcoxon rank-sum test, respectively. Categorical variables were presented in frequency (percentage) and compared using Chi-square or Fisher-exact test.

For patients in* cohort A*, we examined the incidence of ERD over 1 year, between those with CI-AKI versus none. Subsequently, all clinically plausible variables in* cohort A* were included in multivariate binary logistic and linear regression models, to look for independent predictors of CI-AKI/ERD and higher delta-sCr over one year, respectively. In particular,* presence of CI-AKI and delta-sCr *>26 *μ*mol/L at 3 days was included in the model for prediction of ERD. As delta-sCr was not of normal distribution, it was expressed as percentage change from baseline sCr, with addition of a constant (+100), and log-transformed prior to linear regression. The final models were developed using stepwise selection technique, with the *P* value for inclusion being 0.05. A two-sided *P* < 0.05 was taken as measure of statistical significance. Finally, actuarial analysis was performed to illustrate the cumulative incidence of ERD with CI-AKI versus none, in cohort A patients with one or more renal function assessments over subsequent one year, time/day-censored by the latest follow-up sCr measured. Analysis was performed using STATA SE version 13.0 (Lakeway Drive, College Station, Texas, USA).

## 3. Results

**Figure 1:**
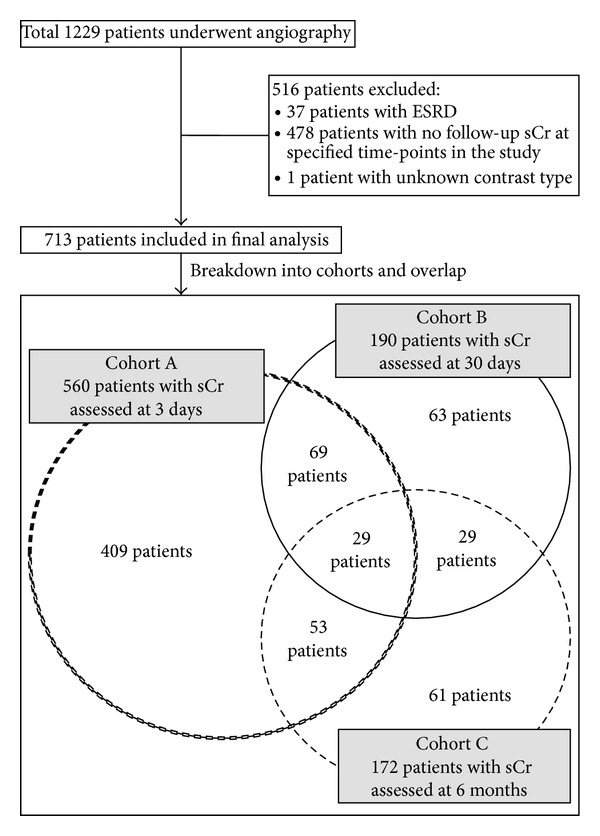
Study flow diagram. ESRD: end-stage renal disease; sCr: serum creatinine.

1229 patients underwent angiography and 713 patients fulfilled the study criteria, with 560, 190, and 172 patients in cohorts A, B, and C, respectively. These cohorts were not mutually exclusive (see Figure 1). Compared to 479 patients* without ESRD* who were excluded, these 713 patients had higher comorbid burden, and more underwent angiography and PCI for emergent indications (see Supplementary Material available online at http://dx.doi.org/10.1155/2014/506479).

Cohort A's profile is shown in Table 1, and profiles of cohorts B and C are shown in supplementary material. In general, patients who received iodixanol (versus iohexol) were older, and more had DM, CCF, STEMI (versus NSTEMI), and concomitant PCI, with higher CM load (corrected for eGFR) (*P* < 0.05). The baseline eGFR of patients given iodixanol versus iohexol in cohorts A, B, and C was 71(±27) versus 88(±26), 64(±27) versus 96(±36), and 65(±27) versus 87(±30) mL/min/1.73 m^2^, respectively (*P* < 0.0001).

For cohort A, 18% had CI-AKI, which was more common in patients given iodixanol (*P* = 0.006). For cohort B, 32% had ERD at 30 days. For cohort C, 34% had ERD at 6 months. 72 patients in cohort C had sCr assessed at 1 year, of which 29 patients (40%) had longer-term ERD. The rates of medium- and longer-term ERD were comparable between patients who received iodixanol or iohexol at baseline.

**Figure 2:**
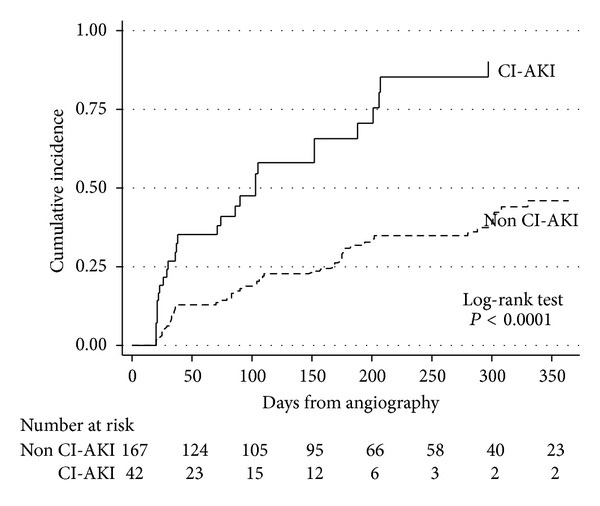
Actuarial analysis of extended renal dysfunction—CI-AKI versus none. CI-AKI: contrast-induced acute kidney injury, defined by RIFLE R/I/F criteria at 3 days after contrast; R/I/F: at-risk/injury/failure; No.: number.

More patients from cohort A with CI-AKI (compared with none) had ERD up to 6 months after angiography (*P* ≤ 0.001) (Table 2). The corresponding incidences of ERD between the presence and absence of CI-AKI at 30 days, 90 days, and 6 months were 67% versus 26%, 75% versus 26%, and 81% versus 26%, respectively (*P* ≤ 0.001) (see Supplementary Material). This association (actuarial analysis) is illustrated in Figure 2, in a subgroup of 209 patients from cohort A with one or more sCr levels assessed over subsequent one year.

On multivariate analysis (Table 3), baseline independent predictors of CI-AKI or higher delta-sCr (at 3-days) included age >65 years, female gender, CCF, STEMI, valvular heart disease or septal defect, IABP insertion, critical illness, PCI, and aortogram. Baseline eGFR <45 mL/min/1.73 m^2^ and CM load >0.7 (per unit eGFR) were associated with* lower* delta-sCr at 3 days. CI-AKI was consistently associated with both medium- and longer-term renal dysfunction. Delta-sCr >26 *μ*mol/L at 3 days was not associated with ERD. Type of CM (iodixanol or iohexol) was not independently associated with CI-AKI or ERD.

## 4. Discussion

### 4.1. Key Findings

The incidence of CI-AKI was 18%. Advanced age, female gender, cardiac comorbidities, STEMI, and critical illness were key predictors of CI-AKI. Contrary to our first hypothesis, the association of CM type with CI-AKI ceased to be significant after adjusting for the above confounders. In accordance with our second hypothesis, CI-AKI was strongly associated with ERD over one year. ERD occurred in up to 30–40% of medium-term survivors who had follow-up renal assessment, and the risk was 3-fold higher in patients with CI-AKI.

### 4.2. Relationship with Previous Studies

CI-AKI is commonly defined by sCr rise >0.5 mg/dL (44 *μ*mol/L) or ≥25% above baseline, and its reported incidence after angiography varies from 3% to >20%, depending on the population at risk [14–16]. Recent studies have classified CI-AKI using RIFLE criteria in patients who underwent coronary interventions. The RIFLE R/I/F incidence ranges from 8 to 16% [17, 18]. Our higher incidence of 18% can be attributed to our inclusion of cardiogenic shock or critical illness and our selected population in whom subsequent renal function assessments were necessary. By redefining CI-AKI from ≥25% to ≥50% rise in sCr using RIFLE criteria, we minimized the chance of overdiagnosis related to conventional definitions, since fluctuations in sCr may still occur in hospitalized patients who do not receive CM [19]. Such numerical fluctuations in sCr may be wider in advanced CKD, and thus defining CI-AKI by relative (versus absolute) change in sCr may be essential. A staging criterion also allows severity grading of CI-AKI. We report a combined RIFLE I/F (≥100% sCr rise) incidence of only 4%, and historically only <1% of patients had severe CI-AKI needing dialysis [20].

Risk predictors of CI-AKI such as advanced age and CCF are consistent with medical literature [16, 21]. Our findings support the higher CI-AKI risk conferred by STEMI and need for IABP, which were previously noted in limited studies [22, 23]. These observations highlight the importance of circulatory failure, cardiogenic shock, and critical illness in influencing acute renal outcomes [24], which are potentially worsened by renal vasoconstriction following CM administration [25]. The inclusion of these emergent conditions in the multivariate model might have diminished the impact of baseline eGFR on CI-AKI. Patients with low baseline eGFR or at higher perceived risk of AKI might have been given prophylactic measures or lower doses of CM, hence explaining the reverse association of low eGFR and CM dose with CI-AKI. Furthermore, the results highlight the specific renal risk posed by STEMI, which is often accompanied by emergent revascularization and hemodynamic disturbance.

In vivo studies have demonstrated that CM induces renal tubular epithelial cell apoptosis [26]. Hyperosmolar solutions contribute to this cytotoxicity, and HOCM induces more renal tubular cell injury than LOCM [27]. In contrast, risk of cytotoxicity from IOCM or LOCM is similar [28], and there is no difference in clinically evident CI-AKI between iodixanol and different LOCMs from recent randomized studies [9, 10, 29]. A higher renal-risk posed by LOCM over IOCM seems to be confined to iohexol in patients with more advanced CKD [8]. In our model, choice of iohexol over iodixanol, or increasing CM load, did not strongly influence early or longer-term renal outcomes, in the presence of other stronger predictors of AKI. These findings support the notion that CI-AKI risk cannot be attributed to contrast osmolarity alone, and other mechanisms such as the clinical context, comorbidities, and hemodynamic condition may play a greater role in AKI pathogenesis.

Using similar modern consensus definitions, 28% of patients from the Alberta registry with mild CI-AKI (≥50% or ≥0.3 mg/dL sCr rise) and 59% with more severe CI-AKI (≥100% sCr rise) had sustained AKI at 3 months, followed by a 0.8 and 2.8 mL/min/1.73 m^2^/year decline in eGFR, respectively [30]. We report that 75% and 81% of patients with CI-AKI had ERD at 3 and 6 months, respectively, in a subcohort of at-risk patients with follow-up sCr measured for clinical indications. More importantly, these patients with CI-AKI, especially those with ERD over months, have a significantly higher risk of long-term mortality, major cardiovascular events, and end-stage renal disease [31, 32]. These observations indicate that CI-AKI may reflect a higher propensity for recurrent renal dysfunction and adverse outcomes. Failure of AKI resolution over time might also suggest alternative disease mechanisms such as cholesterol embolism complicating acute illness and angiography [33].

### 4.3. Clinical Significance of Findings

Our study contributes to growing evidence that CI-AKI is not merely a transient and benign nephropathy but may reflect greater cardiovascular disease burden in high-risk patients [34]. We have identified CI-AKI as a major risk factor for progressive renal deterioration, and this should promote more assiduous follow-up and avoidance of nephrotoxins in these patients. The fact that only a minority of CI-AKI cases fulfilled the more severe RIFLE I/F criteria may be reassuring to clinicians. Finally, the understanding that CM type (iodixanol or iohexol) has less impact on acute or chronic renal outcomes in comparison to more important clinical variables might influence clinicians to reconsider their choice (or avoidance) of respective CM.

### 4.4. Strengths and Limitations

We have evaluated not just acute but extended renal outcomes over one year, in relation to multiple key clinical and procedural factors. The contribution of emergent coronary intervention and critical illness to CI-AKI had so far been assessed by limited studies, and we have demonstrated that these factors are more crucial than CM types in influencing clinical outcomes. This makes our study relevant to daily acute nephrology, cardiology, and radiology practice. We used the RIFLE criteria to classify CI-AKI, an established consensus system, well validated against patient outcomes. The impact of CI-AKI on ERD was strong, and key predictors of renal dysfunction appear logical, plausible, and consistent with expectations. However, iodixanol was used preferentially (over iohexol) in patients perceived at higher risk of AKI due to comorbidities including DM, CCF, CKD, and STEMI; patients with follow-up sCr measurements were naturally selected as individuals at-risk. These constitute selection bias, and the latter implies over-inflated ERD incidence over time. But these mirror actual clinical practice and the results should remain relevant to physicians. Our study was single-centre and observational in nature and all associations cannot be inferred to have causal relationships. We were unable to examine differential outcomes according to various stages of RIFLE criteria, due to low patient numbers with RIFLE I/F. The retrospective nature and variable time window of available sCr also make it impossible to examine the 48-hour sCr change required of the AKIN criteria, which might be more sensitive in AKI diagnosis. We also did not have details of CI-AKI needing dialysis that is otherwise of clinical importance.

## 5. Conclusions

CI-AKI after angiography was common in an elderly patient cohort with significant cardiovascular disease burden, but the majority of cases were mild. The use of iodixanol versus iohexol or CM load has very limited or no positive association with renal outcomes, compared with advanced age, emergent cardiac conditions, cardiogenic shock, and critical illness. CI-AKI is strongly associated with extended renal dysfunction over one year. Our study calls for further prospective research on extended renal outcomes of patients with CI-AKI over several years.

## Supplementary Material

Supplementary table 1 compares the baseline profile of 713 patients included in the final analysis, with 479 non-ESRD patients who met the exclusion criteria. Supplementary tables 2 and 3 show the patient profiles and rates of extended renal dysfunction in cohort B and C, respectively. Supplementary table 4 summarizes the rates of extended renal dysfunction at various time-windows over 1 year, in patients with CI-AKI versus none.

## Figures and Tables

**Table 1:** Baseline profile and short-term impact on renal function (cohort A).

Variables	Cohort A	Iodixanol (Visipaque)	Iohexol (Omnipaque)	*P* value
	*n* = 560	*n* = 297	*n* = 263	
Age, mean (SD), years	65.1 (12.1)	67.4 (12.0)	62.5 (11.8)	<0.0001
Age > 65 years, No. (%)	282 (50.4)	177 (59.6)	105 (39.9)	<0.001
Male gender, No. (%)	389 (69.5)	202 (68.0)	187 (71.1)	0.43
Comorbidities, No. (%)				
Diabetes mellitus	93 (16.6)	60 (20.2)	33 (12.6)	0.02
Hypertensive heart disease	337 (60.2)	184 (62.0)	153 (58.2)	0.36
CCF	59 (10.5)	39 (13.1)	20 (7.6)	0.03
Critical illness (within 1 wk after contrast)	53 (9.5)	30 (10.1)	23 (8.8)	0.58
Baseline renal function				
Serum Cr, median (IQR), *μ*mol/L	81 (68–98)	86 (72–110)	75 (63–86)	<0.0001
eGFR∗, mean (SD), mL/min/1.73 m^2^	79 (28)	71 (27)	88 (26)	<0.0001
eGFR∗ < 60 mL/min/1.73 m^2^, No. (%)	138 (24.6)	110 (37.0)	28 (10.7)	<0.001
eGFR∗ < 45 mL/min/1.73 m^2^, No. (%)	57 (10.2)	46 (15.5)	11 (4.2)	<0.001
Primary cardiac disease, No. (%)				
Suspect CAD (angina or CAD NOS)	172 (30.7)	79 (26.6)	93 (35.4)	0.03
STEMI	113 (20.2)	91 (30.6)	22 (8.4)	<0.001
NSTEMI	176 (31.4)	70 (23.6)	106 (40.3)	<0.001
Arrhythmias	26 (4.6)	12 (4.0)	14 (5.3)	0.47
Valvular heart disease/septal defects	13 (2.3)	9 (3.0)	4 (1.5)	0.27
Cardiomyopathy	17 (3.0)	11 (3.7)	6 (2.3)	0.33
Noncardiac issues	9 (1.6)	7 (2.4)	2 (0.8)	0.18
Others	34 (6.1)	18 (6.1)	16 (6.1)	0.99
Contrast load, median (IQR)				
Contrast volume, mL	150 (100–230)	160 (100–240)	145 (100–210)	0.80
Iodine content, g	51 (34–77)	51 (32–77)	51 (35–74)	0.14
Iodine : eGFR ratio, g per mL/min/1.73 m^2^	0.66 (0.44–1.02)	0.73 (0.47–1.13)	0.60 (0.42–0.91)	0.006
Iodine : eGFR ratio > 0.7	251 (44.8)	150 (50.5)	101 (38.4)	0.004
Procedure details, No. (%)				
Coronary angiogram	551 (98.4)	291 (98.0)	260 (98.9)	0.51
PCI	278 (49.6)	163 (54.9)	115 (43.7)	0.008
Aortogram	34 (6.1)	17 (5.7)	17 (6.5)	0.71
LVgram	365 (65.2)	191 (64.3)	174 (66.2)	0.65
IABP	7 (1.3)	5 (1.7)	2 (0.8)	0.46
Renal function at 3 days after contrast				
Peak sCr, median (IQR), *μ*mol/L	87 (73–106)	96 (81–121)	78 (67–90)	<0.0001
Median day of peak sCr	1 (1-2)	1 (1-2)	1 (1-2)	0.13
ΔCr, median (IQR), *μ*mol/L	4 (−2–16)	8 (−2–20)	1 (−3–10)	0.001
RIFLE “R/I/F”^#^, No. (%)	99 (17.7)	65 (21.9)	34 (12.9)	0.006
RIFLE “I/F”^#^, No. (%)	21 (3.8)	14 (4.7)	7 (2.7)	0.20

∗4-variable MDRD eGFR equation; ^#^RIFLE acute kidney injury classification (“R/I/F” refers to “at-risk/injury/failure” classes, respectively).

Δ: delta (change in); CAD: coronary artery disease; CCF: congestive cardiac failure; D: day; eGFR: estimated glomerular filtration rate; IABP: intra-aortic balloon pump; IQR: interquartile range; LVgram: left ventriculogram; No.: number; NOS: not otherwise specified; NSTEMI: non-ST elevation myocardial infarction; PCI: percutaneous coronary intervention; pts: patients; sCr: serum creatinine; SD: standard deviation; STEMI: ST elevation myocardial infarction; wk: week.

**Table 2:** Extended renal outcomes in patients (cohort A) with CI-AKI versus none.

Total 560 patients (cohort A)	Non-CI-AKI	CI-AKI	*P* value
	*n* = 461	*n* = 99	
Age > 65 years, No. (%)	218 (47.3)	64 (64.7)	0.002
Male gender, No. (%)	334 (72.5)	55 (55.6)	0.001
Comorbidities, No. (%)			
Diabetes mellitus	70 (15.2)	23 (23.2)	0.05
Hypertensive heart disease	281 (61.0)	56 (56.6)	0.42
CCF	34 (7.4)	25 (25.3)	<0.001
Critical illness (within 1 week after contrast)	28 (6.1)	25 (25.3)	<0.001
Baseline renal function			
eGFR∗ < 60 mL/min/1.73 m^2^, No. (%)	120 (26.0)	18 (18.2)	0.10
eGFR∗ < 30 mL/min/1.73 m^2^, No. (%)	45 (9.8)	12 (12.1)	0.48
Iohexol (vs iodixanol), No. (%)	229 (49.7)	34 (34.3)	0.006
Iodine dose per unit eGFR, median (IQR)	0.68 (0.45–1.04)	0.60 (0.40–0.89)	0.06
Iodine dose per unit eGFR > 0.7, No. (%)	214 (46.4)	37 (37.4)	0.10
ΔCr at 3-days, median (IQR), *μ*mol/L	0 (−5–9)	31 (24–55)	<0.0001
Extended renal outcomes			
At 6-months (*n* = 82)	*n* = 66	*n* = 16	
ΔCr, median (IQR), *μ*mol/L	10 (−1–27)	39 (25–45)	0.0005
RIFLE “R/I/F”^#^, No. (%)	17 (25.8)	13 (81.3)	<0.001
RIFLE “I/F”^#^, No. (%)	3 (4.5)	2 (12.5)	0.25
At 1-year (*n* = 80)	*n* = 72	*n* = 8	
ΔCr, median (IQR), *μ*mol/L	9 (−5–32)	17 (6–43)	0.23
RIFLE “R/I/F”^#^, No. (%)	21 (29.2)	4 (50.0)	0.25
RIFLE “I/F”^#^, No. (%)	7 (9.7)	2 (25.0)	0.22

∗4-variable MDRD eGFR equation; ^#^RIFLE acute kidney injury classification (“R/I/F” refers to “at-risk/injury/failure” classes, respectively).

Δ: delta (change in); eGFR: estimated glomerular filtration rate; IQR: interquartile range; No.: number; pts: patients; sCr: serum creatinine; SD: standard deviation; vs: versus.

**Table 3:** Multivariate analysis of renal dysfunction over one year after angiography (cohort A).

Significant variables	Multivariate linear regression			Multivariate logistic regression		
	(log-transformed %ΔCr + constant as dependent variable^†^)			(RIFLE “R/I/F” as dependent variable)		
	Coefficient	95% CI	*P* value	Odds ratio	95% CI	*P* value
Renal dysfunction at 3 days after contrast						
Age > 65 yrs (Y/N)	0.05	0.01–0.09	0.02	1.88	1.11–3.21	0.02
Male gender (vs female)	−0.05	−0.09–−0.01	0.02	0.40	0.24–0.67	0.001
CCF (Y/N)	0.07	0.01–0.14	0.03	2.62	1.35–5.08	0.004
Baseline eGFR < 45	−0.09	−0.15–−0.02	0.009			
STEMI (Y/N)	0.07	0.02–0.12	0.007	2.08	1.11–3.89	0.02
Valvular HD or septal defect (Y/N)	0.14	0.02–0.27	0.03	7.67	2.18–27.04	0.002
Critical illness (Y/N)	0.21	0.14–0.27	<0.001	4.45	2.18–9.08	<0.001
IABP (Y/N)	0.43	0.25–0.62	<0.001	17.66	1.80–173.55	0.01
PCI (Y/N)	0.05	0.00–0.09	0.04			
Aortogram (Y/N)	0.08	0.00–0.16	0.04			
Iodine dose per unit eGFR > 0.7	−0.08	−0.12–−0.04	<0.001	0.50	0.29–0.86	0.01
Renal dysfunction at 30 days after contrast∗						
CI-AKI (Y/N)	0.30	0.13–0.47	0.001	12.75	2.31–70.50	0.004
Baseline eGFR < 45				0.05	0.00–0.80	0.04
Hypertensive HD (Y/N)	0.16	0.01–0.30	0.03	3.82	1.05–13.97	0.04
Valvular HD or septal defect (Y/N)	0.30	0.04–0.57	0.03			
Aortogram (Y/N)				16.45	1.81–149.51	0.01
Iodine dose per unit eGFR > 0.7	−0.20	−0.34–−0.06	0.007	0.08	0.02–0.33	0.001
Renal dysfunction at 6 months after contrast∗						
CI-AKI (Y/N)	0.39	0.26–0.52	<0.001	15.31	3.03–77.35	0.001
Suspect CAD without AMI (Y/N)	0.18	0.06–0.30	0.003			
IABP (Y/N)	−0.74	−1.24–−0.25	0.004			
PCI (Y/N)	−0.18	−0.30–−0.07	0.003			
Aortogram (Y/N)	0.25	0.05–0.45	0.01			
Renal dysfunction at 1 year after contrast∗						
CI-AKI (Y/N)	0.28	0.02–0.53	0.03			

Variables included in multivariate models include baseline variables: age > 65 yrs; male gender (vs females); DM (y/n); hypertensive HD (y/n); CCF (y/n); baseline eGFR < 45 mL/min/1.73 m^2^; suspect CAD without AMI (y/n); STEMI (y/n); NSTEMI (y/n); arrhythmias (y/n); valvular HD or septal defect (y/n); CMP (y/n) and periprocedure variables: COROS (y/n); aortogram (y/n); PCI (y/n); LVgram (y/n); IABP (y/n); iohexol use (vs iodixanol); iodine dose per unit eGFR > 0.7; critical illness within 1 wk after contrast (y/n).

∗Additional variables added to model: CI-AKI (y/n); ΔCr > 26 *μ*mol/L (0.3 mg/dL) at 3 days after contrast.

^†^Delta-Cr expressed as % change from baseline Cr, with addition of constant (100), and log-transformed prior to linear regression.

ΔCr: (delta) change in Cr; AMI: acute myocardial infarction; CAD: coronary artery disease; CCF: congestive cardiac failure; CI: confidence interval; CMP: cardiomyopathy; COROS: coronary angiogram; Cr: serum creatinine; DM: diabetes mellitus; eGFR: estimated glomerular filtration rate; HD: heart disease; IABP: intra-aortic balloon pump; LVgram: left ventriculogram; NSTEMI: non-STEMI; PCI: percutaneous coronary intervention; STEMI: ST elevation myocardial infarction; vs: versus; wk: week; yrs: years.
