# Supplementary material for: Extended Renal Outcomes with Use of Iodixanol versus Iohexol after Coronary Angiography
Source: Biomed Res Int. 2014 Aug 7;2014:506479. doi: 10.1155/2014/506479 (PMC4142278; doi:10.1155/2014/506479)
Supplement: Supplementary file 1 — Supplementary table 1 compares the baseline profile of 713 patients included in the final analysis, with 479 non-ESRD patients who met the exclusion criteria. Supplementary tables 2 and 3 show the patient profiles and rates of extended renal dysfunction in cohort B and C, respectively. Supplementary table 4 summarizes the rates of extended renal dysfunction at various time-windows over 1 year, in patients with CI-AKI versus none. [file 506479.f1.doc]

***R1 – Supplementary tables***

**EXTENDED RENAL OUTCOMES WITH USE OF IODIXANOL VERSUS IOHEXOL AFTER CORONARY ANGIOGRAPHY**

Horng-Ruey CHUA^1,3^, Mark HORRIGAN^2^, Elizabeth MCINTOSH^2^, Rinaldo BELLOMO^1,4^

^1^*Department of Intensive Care, Austin Hospital, Melbourne, Australia*

^2^*Department of Cardiology, Austin Hospital, Melbourne, Australia*

^3^*Division of Nephrology, University Medicine Cluster, National University Hospital, National University Health System, Singapore*

^4^*Australian and New Zealand Intensive Care Research Committee (ANZIC-RC), Monash University, School of Public Health and Preventive Medicine, Melbourne, Australia*

**Corresponding author**

Professor Rinaldo Bellomo

Director of Intensive Care Research, Austin Hospital, Melbourne, Australia

Co-director of Australian and New Zealand Intensive Care Research Centre (ANZIC-RC),

Monash University, Commercial Road, Melbourne, Victoria 3181, Australia

Tel: +61-3-9496 5992; Fax: +61-3-9496 3932

E-mail: [rinaldo.bellomo@austin.org.au](mailto:rinaldo.bellomo@austinrmc.org.au)

| **SUPPLEMENTARY TABLE 1: Profile of patients excluded from final analysis*** | | | | | | |
| --- | --- | --- | --- | --- | --- | --- |
| **Variables** | | **Fulfilled study criteria** | | **Excluded patients*** | | **p-value** |
|  |  | **n = 713** | | **n = 479** | |  |
| **Age >65 years, No. (%)** | | 363 | (50.9) | 235 | (49.1) | 0.53 |
| **Male gender, No. (%)** | | 495 | (69.4) | 307 | (64.1) | 0.05 |
| **Co-morbidities, No. (%)** | |  |  |  |  |  |
|  | ***Diabetes mellitus*** | 98 | (13.8) | 42 | (8.8) | 0.01 |
|  | ***Hypertensive heart disease*** | 389 | (54.6) | 147 | (31.0) | <0.001 |
|  | ***CCF*** | 60 | (8.4) | 12 | (2.5) | <0.001 |
|  | ***Critical illness (within 1 wk post-contrast)*** | 55 | (7.7) | 2 | (0.7) | <0.001 |
| **Primary cardiac disease, No. (%)** | |  |  |  |  |  |
|  | **Suspect CAD (angina or CAD NOS)** | 279 | (39.1) | 377 | (78.7) | <0.001 |
|  | **STEMI** | 114 | (16.0) | 7 | (1.5) | <0.001 |
|  | **NSTEMI** | 177 | (24.8) | 25 | (5.2) | <0.001 |
|  | **Arrhythmias** | 26 | (3.7) | 7 | (1.5) | 0.02 |
|  | **Valvular heart disease / septal defects** | 33 | (4.6) | 9 | (1.9) | 0.01 |
|  | **Cardiomyopathy** | 22 | (3.1) | 18 | (3.8) | 0.53 |
| **Procedure details, No. (%)** | |  |  |  |  |  |
|  | **Coronary angiogram** | 700 | (98.2) | 468 | (97.7) | 0.57 |
|  | **PCI** | 279 | (39.1) | 34 | (7.1) | <0.001 |
|  | **Aortogram** | 53 | (7.4) | 42 | (8.8) | 0.40 |
|  | **LVgram** | 493 | (69.1) | 409 | (85.4) | <0.001 |
|  | **IABP** | 7 | (1.0) | 1 | (0.2) | 0.15 |
| *: excluding patients with baseline end-stage renal disease; CAD: coronary artery disease; CCF: congestive cardiac failure; IABP: intra-aortic balloon pump; LVgram: left ventriculogram; No.: number; NOS: not otherwise specified; NSTEMI: non-ST elevation myocardial infarction; PCI: percutaneous coronary intervention; SD: standard deviation; STEMI: ST elevation myocardial infarction; wk: week. | | | | | | |

| **SUPPLEMENTARY TABLE 2: Medium-term impact on renal function (cohort B)** | | | | | | | | |
| --- | --- | --- | --- | --- | --- | --- | --- | --- |
| **Variables** | | **Cohort B** | | **Iodixanol (Visipaque)** | | **Iohexol (Omnipaque)** | | **p-value** |
|  |  | **n = 190** | | **n = 93** | | **n = 97** | |  |
| **Age, mean (SD), years** | | 65.9 | (12.1) | 68.8 | (11.0) | 63.1 | (12.5) | 0.0005 |
| **Age >65 years, No. (%)** | | 104 | (54.7) | 62 | (66.7) | 42 | (43.3) | 0.001 |
| **Male gender, No. (%)** | | 129 | (67.9) | 59 | (63.4) | 70 | (72.2) | 0.20 |
| **Co-morbidities, No. (%)** | |  |  |  |  |  |  |  |
|  | ***Diabetes mellitus*** | 31 | (16.4) | 23 | (24.7) | 8 | (8.3) | 0.002 |
|  | ***Hypertensive heart disease*** | 90 | (47.4) | 49 | (52.7) | 41 | (42.3) | 0.15 |
|  | ***CCF*** | 18 | (9.5) | 14 | (15.1) | 4 | (4.1) | 0.01 |
|  | ***Critical illness (within 1 wk post-contrast)*** | 15 | (7.9) | 9 | (9.7) | 6 | (6.2) | 0.37 |
| **Baseline renal function** | |  |  |  |  |  |  |  |
|  | ***Serum Cr, median (IQR), μmol/L*** | 80 | (67-101) | 95 | (73-117) | 71 | (62-86) | <0.0001 |
|  | ***eGFR*, mean (SD), ml/min/1.73m^2^*** | 80 | (35) | 64 | (27) | 96 | (36) | <0.0001 |
|  | ***eGFR* < 60 ml/min/1.73m^2^, No. (%)*** | 53 | (27.9) | 42 | (45.2) | 11 | (11.3) | <0.001 |
|  | ***eGFR* < 45 ml/min/1.73m^2^, No. (%)*** | 22 | (11.6) | 20 | (21.5) | 2 | (2.1) | <0.001 |
| **Contrast load, median (IQR)** | |  |  |  |  |  |  |  |
|  | **Contrast volume, ml** | 120 | (80-230) | 120 | (80-235) | 113 | (80-210) | 0.92 |
|  | **Iodine content, g** | 39 | (27-75) | 38 | (26-75) | 39 | (28-74) | 0.29 |
|  | **Iodine:eGFR ratio, g per ml/min/1.73m^2^** | 0.56 | (0.36-0.97) | 0.63 | (0.41-1.11) | 0.52 | (0.32-0.81) | 0.003 |
|  | **Iodine:eGFR ratio > 0.7** | 71 | (37.4) | 42 | (45.2) | 29 | (29.9) | 0.03 |
| **Renal function at 30 days post contrast** | |  | |  | |  | |  |
|  | ***Peak sCr, median (IQR), μmol/L*** | 93 | (75-121) | 104 | (86-139) | 83 | (71-105) | <0.0001 |
|  | ***Median day of peak sCr*** | 29 | (22-34) | 29 | (22-34) | 29 | (22-34) | 0.79 |
|  | ***Δ Cr, median (IQR), μmol/L*** | 11 | (0-27) | 11 | (0-27) | 10 | (2-24) | 0.88 |
|  | ***30-day RIFLE "R/I/F"*^╫^*, No. (%)*** | 60 | (31.6) | 29 | (31.2) | 31 | (32.0) | 0.91 |
|  | ***30-day RIFLE "I/F"*^╫^*, No. (%)*** | 17 | (9.0) | 7 | (7.5) | 10 | (10.3) | 0.50 |
|  |  |  |  |  |  |  |  |  |
| **Renal function at 90-days post contrast**** | | **n = 82** | | **n = 44** | | **n = 38** | |  |
|  | ***Peak sCr, median (IQR), μmol/L*** | 101 | (78-150) | 124 | (89-200) | 87 | (74-109) | 0.001 |
|  | ***Median day of peak sCr*** | 84 | (76-97) | 85 | (76-98) | 84 | (75-95) | 0.77 |
|  | ***Δ Cr, median (IQR), μmol/L*** | 18 | (1-48) | 19 | (0-71) | 16 | (6-31) | 0.76 |
|  | ***90-day RIFLE "R/I/F"*^╫^*, No. (%)*** | 36 | (43.9) | 20 | (45.5) | 16 | (42.1) | 0.76 |
|  | ***90-day RIFLE "I/F"*^╫^*, No. (%)*** | 16 | (19.5) | 10 | (22.7) | 6 | (15.8) | 0.43 |
| *: 4-variable MDRD eGFR equation; ^╫^: RIFLE acute kidney injury classification ("R/I/F" refers to "at risk/injury/failure" classes, respectively); **: refers to subgroup of patients with available Cr done at D70-D110 post-contrast. | | | | | | | | |
| Δ: delta (change in); CCF: congestive cardiac failure; eGFR: estimated glomerular filtration rate; IQR: interquartile range; No.: number; pts: patients; sCr: serum creatinine; SD: standard deviation. | | | | | | | | |

| **SUPPLEMENTARY TABLE 3: Longer-term impact on renal function (cohort C)** | | | | | | | | |
| --- | --- | --- | --- | --- | --- | --- | --- | --- |
| **Variables** | | **Cohort C** | | **Iodixanol (Visipaque)** | | **Iohexol (Omnipaque)** | | **p-value** |
|  |  | **n = 172** | | **n = 83** | | **n = 89** | |  |
| **Age, mean (SD), years** | | 65.0 | (11.9) | 65.8 | (11.7) | 64.2 | (12.1) | 0.19 |
| **Age >65 years, No. (%)** | | 93 | (54.1) | 49 | (59.0) | 44 | (49.4) | 0.21 |
| **Male gender, No. (%)** | | 115 | (66.9) | 53 | (63.9) | 62 | (69.7) | 0.42 |
| **Co-morbidities, No. (%)** | |  |  |  |  |  |  |  |
|  | ***Diabetes mellitus*** | 18 | (10.5) | 12 | (14.5) | 6 | (6.7) | 0.10 |
|  | ***Hypertensive heart disease*** | 67 | (39.0) | 40 | (48.2) | 27 | (30.3) | 0.02 |
|  | ***CCF*** | 12 | (7.0) | 6 | (7.2) | 6 | (6.7) | 0.90 |
|  | ***Critical illness (within 1 wk post-contrast)*** | 10 | (5.8) | 4 | (4.8) | 6 | (6.7) | 0.75 |
| **Baseline renal function** | |  |  |  |  |  |  |  |
|  | ***Serum Cr, median (IQR), μmol/L*** | 82 | (69-102) | 92 | (74-121) | 75 | (65-88) | <0.0001 |
|  | ***eGFR*, mean (SD), ml/min/1.73m^2^*** | 77 | (30) | 65 | (27) | 87 | (30) | <0.0001 |
|  | ***eGFR* < 60 ml/min/1.73m^2^, No. (%)*** | 52 | (30.2) | 38 | (45.8) | 14 | (15.7) | <0.001 |
|  | ***eGFR* < 45 ml/min/1.73m^2^, No. (%)*** | 23 | (13.4) | 20 | (24.1) | 3 | (3.4) | <0.001 |
| **Contrast load, median (IQR)** | |  |  |  |  |  |  |  |
|  | **Contrast volume, ml** | 100 | (80-150) | 100 | (75-175) | 100 | (80-138) | 0.94 |
|  | **Iodine content, g** | 34 | (26-53) | 32 | (24-56) | 35 | (28-48) | 0.28 |
|  | **Iodine:eGFR ratio, g per ml/min/1.73m^2^** | 0.52 | (0.36-0.88) | 0.61 | (0.41-1.08) | 0.47 | (0.34-0.64) | 0.003 |
|  | **Iodine:eGFR ratio > 0.7** | 47 | (27.3) | 30 | (36.1) | 17 | (19.1) | 0.01 |
| **Renal function at 6 months post contrast** | |  | |  | |  | |  |
|  | ***Peak sCr, median (IQR), μmol/L*** | 94 | (78-130) | 114 | (85-162) | 86 | (75-111) | 0.0001 |
|  | ***Median day of peak sCr*** | 179 | (161-199) | 178 | (161-198) | 179 | (159-201) | 0.98 |
|  | ***Δ Cr, median (IQR), μmol/L*** | 12 | (0-31) | 14 | (0-40) | 8 | (0-27) | 0.24 |
|  | ***6 month RIFLE "R/I/F"*^╫^*, No. (%)*** | 59 | (34.3) | 28 | (33.7) | 31 | (34.8) | 0.88 |
|  | ***6 month RIFLE "I/F"*^╫^*, No. (%)*** | 17 | (9.9) | 8 | (9.6) | 9 | (10.1) | 0.92 |
|  |  |  |  |  |  |  |  |  |
| **Renal function at 1 year post contrast**** | | **n = 72** | | **n = 38** | | **n = 34** | |  |
|  | ***Peak sCr, median (IQR), μmol/L*** | 112 | (85-153) | 116 | (86-189) | 106 | (84-148) | 0.56 |
|  | ***Median day of peak sCr*** | 310 | (289-351) | 315 | (290-362) | 307 | (287-345) | 0.47 |
|  | ***Δ Cr, median (IQR), μmol/L*** | 20 | (0-48) | 15 | (-3 - 36) | 24 | (5-78) | 0.26 |
|  | ***1 year RIFLE "R/I/F"*^╫^*, No. (%)*** | 29 | (40.3) | 12 | (31.6) | 17 | (50.0) | 0.11 |
|  | ***1 year RIFLE "I/F"*^╫^*, No. (%)*** | 15 | (20.8) | 6 | (15.8) | 9 | (26.5) | 0.27 |
| *: 4-variable MDRD eGFR equation; ^╫^: RIFLE acute kidney injury classification ("R/I/F" refers to "at risk/injury/failure" classes, respectively); **: refers to subgroup of patients with available Cr done at D270-D450 post-contrast. | | | | | | | | |
| Δ: delta (change in); CCF: congestive cardiac failure; eGFR: estimated glomerular filtration rate; IQR: interquartile range; No.: number; pts: patients; sCr: serum creatinine; SD: standard deviation. | | | | | | | | |

| **SUPPLEMENTARY TABLE 4: Detailed renal outcomes over 1 year, in patients with CI-AKI versus none (from cohort A)** | | | | | | |
| --- | --- | --- | --- | --- | --- | --- |
| **Total 560 patients (cohort A)** | | **Non CI-AKI** | | **CI-AKI** | | **p-value** |
|  |  | **n = 461** | | **n = 99** | |  |
| **Subsequent changes in renal function** | |  | |  | |  |
|  | ***30-day peak sCr (n = 98)*** | **n = 77** | | **n = 21** | |  |
|  | ***Δ Cr, median (IQR), μmol/L*** | 7 | (-4 - 22) | 34 | (16-67) | 0.0001 |
|  | ***RIFLE "R/I/F"*^╫^*, No. (%)*** | 20 | (26.0) | 14 | (66.7) | 0.001 |
|  | ***RIFLE "I/F"*^╫^*, No. (%)*** | 5 | (6.5) | 7 | (33.3) | 0.001 |
|  | ***90-day peak sCr (n = 92)*** | **n = 72** | | **n = 20** | |  |
|  | ***Δ Cr, median (IQR), μmol/L*** | 7 | (-7 - 34) | 30 | (18-66) | 0.002 |
|  | ***RIFLE "R/I/F"*^╫^*, No. (%)*** | 19 | (26.4) | 15 | (75.0) | <0.001 |
|  | ***RIFLE "I/F"*^╫^*, No. (%)*** | 7 | (9.7) | 4 | (20.0) | 0.25 |
|  | ***6-month peak sCr (n = 82)*** | **n = 66** | | **n = 16** | |  |
|  | ***Δ Cr, median (IQR), μmol/L*** | 10 | (-1 - 27) | 39 | (25-45) | 0.0005 |
|  | ***RIFLE "R/I/F"*^╫^*, No. (%)*** | 17 | (25.8) | 13 | (81.3) | <0.001 |
|  | ***RIFLE "I/F"*^╫^*, No. (%)*** | 3 | (4.5) | 2 | (12.5) | 0.25 |
|  | ***1-year peak sCr (n = 80)*** | **n = 72** | | **n = 8** | |  |
|  | ***Δ Cr, median (IQR), μmol/L*** | 9 | (-5 - 32) | 17 | (6-43) | 0.23 |
|  | ***RIFLE "R/I/F"*^╫^*, No. (%)*** | 21 | (29.2) | 4 | (50.0) | 0.25 |
|  | ***RIFLE "I/F"*^╫^*, No. (%)*** | 7 | (9.7) | 2 | (25.0) | 0.22 |
| ^╫^: RIFLE acute kidney injury classification ("R/I/F" refers to "at risk/injury/failure" classes, respectively). | | | | | | |
| Δ Cr: delta (change in) peak sCr at respective time-windows from baseline; IQR: interquartile range; No.: number; sCr: serum creatinine. | | | | | | |
